# Addressing Suicide Risk According to Different Healthcare Professionals in Spain: A Qualitative Study

**DOI:** 10.3390/ijerph15102117

**Published:** 2018-09-26

**Authors:** Juan-Luis Muñoz-Sánchez, María Cruz Sánchez-Gómez, María Victoria Martín-Cilleros, Esther Parra-Vidales, Diego de Leo, Manuel A. Franco-Martín

**Affiliations:** 1 Psychiatry and Mental Health Department, Hospital Universitario Río Hortega, C/ Dulzaina, 2, 47012 Valladolid, Spain; mfrancom@saludcastillayleon.es; 2 Departamento de Didáctica, Organización y Métodos de Investigación, Facultad de Educación, Universidad de Salamanca, Paseo de Canalejas 169, 37008 Salamanca, Spain; mcsago@usal.es (M.C.S.-G.); viquimc@usal.es (M.V.M.-C.); 3 INTRAS Foundation, Ctra. de la Hiniesta 137, 49024 Zamora, Spain; stherpv@gmail.com; 4 Australian Institute for Suicide Research and Prevention, Griffith University, Mt Gravatt Campus, Mount Gravatt, QLD 4122, Australia; d.deleo@griffith.edu.au; 5 Departamento de Personalidad, Evaluación y Tratamiento psicológico, Facultad de Psicología, Universidad de Salamanca, Campus Ciudad Jardín, 37005 Salamanca, Spain

**Keywords:** suicide, suicidal behavior, risk of suicide, suicide prevention, health professionals

## Abstract

This study analyzes the views of four groups of healthcare professionals who may play a role in the management of suicidal behavior. The goal was to identify key factors for suicide prevention in different areas of the healthcare system. Qualitative research was conducted using focus groups made up of different healthcare professionals who participated in the identification, management, and prevention of suicidal behavior. Professionals included were primary care physicians, psychologists, psychiatrists, and emergency physicians. ‘Suicide’ was amongst the most relevant terms that came up in discussions most of the times it appeared associated with words such as ‘risk’, danger’, or ‘harm’. In the analysis by categories, the four groups of professionals agreed that interventions in at-risk behaviors are first in importance. Prevention was the second main concern with greater significance among psychiatrists. Primary care professionals call for more time to address patients at risk for suicide and easier access to and communication with the mental health network. Emergency care professionals have a lack of awareness of their role in the detection of risk for suicide in patients who seek attention at emergency care facilities for reasons of general somatic issues. Mental health care professionals are in high demand in cases of self-harm, but they would like to receive specific training in dealing with suicidal behavior.

## 1. Introduction

Suicide is a serious public health issue and one of the most frequent causes of unnatural death in the world, with approximately 800,000 people dying by it every year in the world [1]. It is one of the leading causes of death among young people, being one of the top three in the 15–44 age range and ranking second in the 15–19 age group [1]. Although the global rate of suicide in Europe is high, its epidemiology differs widely across the countries [2]. Hence, suicide prevention is at the core of the operational program of the World Health Organization, whose aim is to lower suicide rates by 10% by the year 2020 [1]. The first step towards such goal is effective detection. There are a number of suicide risk screening and assessment strategies available to healthcare professionals, researchers, and educators, but no consensus has been reached on establishing a gold standard to detect suicide risk and manage suicidal behavior [3]. Nonetheless, the importance of risk detection in suicide prevention is clear from the fact that 91% of those who lose their lives to suicide have been in touch with healthcare professionals at some point during the year before death, and that 66% are involved in some manner with the mental health network, mainly at outpatient centers [4].

Suicidal behavior is usually influenced by a variety of factors whose nature can be biological, genetic, psychological, social, environmental, or circumstantial [5]. In this regard, suicide and suicidal behavior are closely linked to the kind of society in which the individual lives [6]. Nevertheless, it should be noted that a previous history of suicidal ideation is an important risk factor, and that having attempted suicide is the most relevant predictor of death by suicide [7]. In fact, approximately 60% of the transitions from suicidal ideation to planned or attempted suicide take place in the first year after the onset of such ideation [8]. On the other hand, the existence or history of mental illness is the main risk factor in the general population [9,10,11]; mood disorders, poor impulse-control, alcohol and substance abuse, and psychotic and personality disorders are the ones that carry a higher risk of suicide and suicidal behavior [12,13,14].

Suicidal acts are usually preceded by milder manifestations such as thoughts of death and suicidal ideation [15]. The evolution from thought to act is the transition from mild to severe symptoms in the suicidal process [16]. Suicidal behaviors are one of the leading causes of morbidity and mortality, and are closely linked to affective disorders [17,18]. Suicide rates are generally quite higher in people suffering from mood disorders, while the frequency of attempts is lower, which might indicate a higher risk for death in individuals suffering from affective disorders [19].

A patient’s suicide always has a huge impact on healthcare professionals, especially on those working in the area of mental health, affecting them both at the professional and the personal levels [20]. Indeed, it can increase awareness of the factors involved in suicide risk [21], although, on the other hand, being involved in the care of people at risk for suicide can also trigger rejection, fear, and high levels of stress [22]. In general, healthcare professionals are sufficiently educated about suicidal behavior, but still there are certain lacks and problems that hinder an effective approach to it [23]. However, not all health professionals have the same predisposition and interest in this aspect of public health [24,25]. Moreover, healthcare professionals often display negative attitudes towards patients with suicidal behaviors [24]. Therefore, there is a need to improve the training of health professionals in the field of suicide [6]. In addition, an adequate training in the detection and management of suicide risk is crucial for its prevention [26]. In this regard, there are specific training programs for healthcare professionals to acquire skills in the assessment of suicidal behavior and in crisis intervention that have proved effective, increasing the expertise and self-confidence of these professionals when faced with suicide-related behaviors [27]. This is why many healthcare professionals express the need for training in how to identify signs and symptoms of suicide risk [28], and over half of the mentioned professionals believe that they require preparation to successfully address patients who have already attempted it [29].

Primary care physicians and staff and emergency medicine professionals are those who are most closely in contact with patients at risk or who have performed a suicidal act [30,31,32]. While primary care physicians are front-line in suicidal risk detection [33], they frequently find it hard to identify and assess, which renders the implementation of suicide prevention programs in the area of primary care necessary [34]. On the other hand, emergency physicians usually have problems when it comes to addressing suicidal behavior, reporting time constraints, lack of privacy, difficulties consulting with other professionals, and absence of specific action protocols as the main barriers they face [35]. This is why effective training programs devoted to suicidal behavior and its management are so necessary [36]. Finally, even though psychiatrists and psychologists are in closer contact with individuals at risk for suicide and are trained to bear the weight of the intervention [37], many of them lack training in current best-practice clinical guidelines for suicide risk assessment and crisis management. There is a variability in the approach to suicide according to different mental health professionals and it is a fact that not all professionals execute evidence-based interventions [38,39]. Psychiatrists are the most knowledgeable and experienced professionals about suicide issue; this is why they are considered key professionals in this field [40]. Psychiatrists usually take greater on responsibility in decision making as regards intervention plans for people with suicidal behavior [41]. Accordingly, psychiatrists use to have a more realistic view approaching to the problem of suicide and regarding the development of prevention programs, but they are also the most aware professionals of the existing limitations [42]. An example of these limitations would be the fact that a large number of suicides are not preceded by a change in the patient’s clinical conditions, and this situation implies a serious handicap in order to identify the risk of suicide [43]. Psychologists are another professional category with a leading role in the field of suicide, both in prevention and in follow-up care in family environment [44,45]. Psychologists, for their part, are more concerned with the identification and treatment of the earliest signs and symptoms of risk for suicide, as well as with the prevention and eradication of risk behaviors in patients who have already attempted suicide [46,47,48]. However, it should be emphasized that these professionals could have an even greater implication in the treatment of people at risk of suicide [49].

The purpose of this study is to analyze the views of four groups of healthcare professionals who play a relevant role in the management of suicide risk and related behaviors with the goal of identifying the key factors for suicide prevention in different areas of the healthcare system. Our objective is the use of the results of this qualitative research for the creation of a needs study which allows the implementation, in our health area, specific training groups of different professional cadres involved in the approach, treatment and prevention of the suicidal behaviors. This research is part of the European Regions Enforcing Actions Against Suicide (EUREGENAS) European project, which brings together 11 regions with different experiences with the aim to contribute to suicide prevention in Europe [50,51].

## 2. Materials and Methods

### 2.1. Design

Qualitative research was conducted using focus groups made up of different healthcare professionals who participated in the identification, management, and prevention of suicidal behavior. The study was carried out in the context of the EUREGENAS project.

### 2.2. Inclusion Criteria

A total of 56 participants were recruited based on the following inclusion criteria:

- Healthcare professional belonging to one of the four groups selected for the study: psychiatrists, psychologists, primary care physicians, and emergency medicine physicians.
- Professional experience in the area of suicide.
- Age between 18 and 65 years.

### 2.3. Recruitment

Participants were recruited from different centers of the INTRAS Foundation and from different healthcare units of the province of Zamora (Spain), which was where the trial was conducted. With regard to sex, 70.6% of the participants were women and 29.4% were men. The average age of the participants was 41, and the average number of years of professional experience was 14.

Recruitment was carried out through purposive sampling, thus preventing generalization in terms of probability, and managing to register the variety of opinions on suicide prevention among the different health professionals to create as much discursive space as possible. 

This deliberate sampling included healthcare professionals in the areas involved in the prevention of suicidal behavior: primary care physicians (primary care network), psychologists/psychiatrists (mental health network), and emergency medicine physicians (emergency care network). Broadly speaking, the primary care network plays a relevant role in detecting the risk for suicide, emergency care handles suicidal behavior, which is usually an urgent matter, and, finally, mental health professionals intervene in the reduction or eradication of the risk for suicide.

### 2.4. Procedure

The description and understanding of the experiences, perspectives, opinions, and meanings expressed by the health professionals that are in closest contact with suicide issues in terms of detection, management, and treatment of suicide-related behaviors was carried out using qualitative methods. This methodological experience grants access to reality without the need for previous categorization. Participants were allowed to express themselves spontaneously in natural contexts, yielding significant research results in the area of psychiatry [52,53] and, more specifically, in the matter of suicide [33,54,55]. Inter- and intra-subject information gathering was conducted using a group interview (focus group) technique, which requires participants’ involvement and provides insight into their subjective scenario.

**Figure 1:**
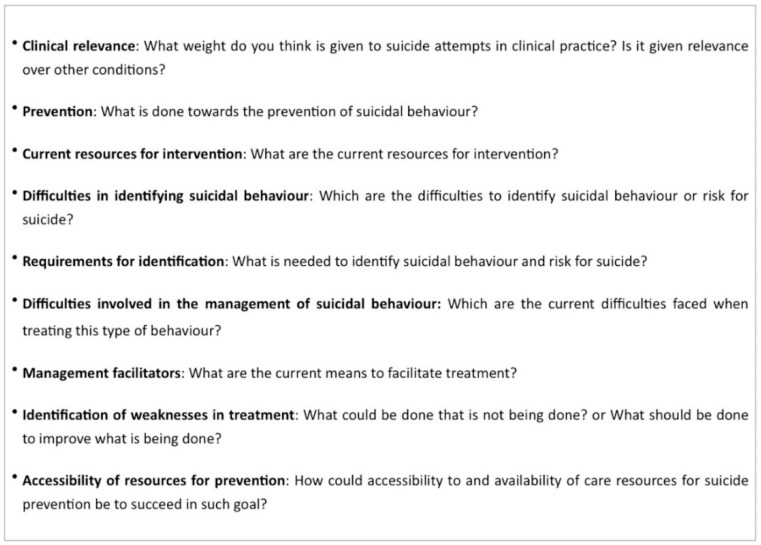
Thematic script for the healthcare professionals’ focus group sessions.

Participants were distributed into eight focus groups (two for each professional category), made up of 12 primary care physicians, 14 emergency physicians, 17 psychologists, and 13 psychiatrists. The groups were structured into strata and balanced according to the socio-demographic characteristics of the participants of each professional specialty. Focus group sessions lasted between 1 and 1.5 h and were audio and video recorded. To ensure greater objectivity, the sessions were conducted by two expert researchers in qualitative dynamics from the University of Salamanca who had no background knowledge of suicide (Sanchez-Gomez, M.C.; Martin-Cilleros, M.V.). The interviews were carried out using a script of open-ended questions drawn up in agreement with expert researchers in the mental health area (Munoz-Sanchez, J.L.; Parra-Vidales, E.; Franco-Martin, M.A) who, acting as a panel of experts, made it possible to identify the most relevant aspects in approaching, treating, and preventing suicide-related behavior (Figure 1). The goal was to avoid guided interviews where questions might hint at a desired response. Before starting the interview, and with the prior approval of the relevant ethics committee, participants signed the informed consent form and filled out a socio-demographic questionnaire to make subsequent sample characterization possible. Meetings flowed smoothly and in a very participative atmosphere, which encouraged subjects to speak freely, expressing their ideas individually and interactively. The meetings were an attempt to describe and interpret the inter- and intra-professional differences that make it possible to differentiate the meaning of suicidal behavior prevention for each professional group.

### 2.5. Analysis

The material obtained from focus group recording was transcribed and the generated script was coded. All the speech produced, freely and spontaneously, was considered relevant. Classical qualitative content analysis was used for textual data processing with the support of Nvivo 10 software (© QSR International Pty Ltd, Melbourne, Australia). The qualitative content analysis is defined as a “research method for the subjective interpretation of the content of text data through the systematic classification process of coding and identifying themes or patterns” [56]. In our research, an inductive content analysis (or conventional content analysis) has been used for the analysis of focus group discussions. In inductive content analysis coding categories are derived directly and inductively from the raw data. Researchers avoid using preconceived categories, allowing the categories and names for categories to “flow from the data” instead [56]. The advantage of the conventional approach of content analysis is that direct information is gained from the study participants without preconceived theoretical perspectives having been imposed [57].

The steps followed were those of a basic analytical process, used in most of the research conducted with this type of data: (a) data transcription; (b) data layout and processing; (c) drawing of results and verification of findings. It should be noted that in qualitative research these stages may overlap, since the design of qualitative research is emergent.

The analysis developed as follows: transcription of group interviews, categorization or transformation of text into data, and, finally, coding or allocation of a textual space to the corresponding category of the information gathered.

**Figure 2:**
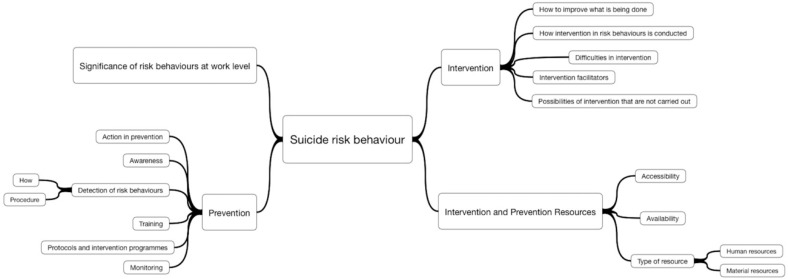
Main categories and subcategories of suicide risk behavior significance.

Categorization is the process in which ideas and objects are recognized, differentiated, and understood. Categorization implies that objects are grouped into categories. In this study an in vivo, open and axial coding was done to obtain the central concepts. Thus, a categories concept map was produced (Figure 2) according to the goals of the study, the protocol questions and the ideas expressed by the participants on aspects related to suicidal behavior. The most representative dimensions or ideas were outlined and arranged hierarchically into 4 categories or main axes and 14 subcategories. Categorization was carried out following the criteria of quality, thoroughness, significance, accuracy, replicability, and exclusivity. Coding was conducted under the supervision of several experts in qualitative research from the University of Salamanca and of a group of mental health experts, thus ensuring credibility, dependence (reliability), and confirmability (objectivity) of the analysis process.

## 3. Results

The qualitative analyses were conducted as follows: first, the most representative words and their meaning in the healthcare context were described to subsequently offer a profile of the main categories (coding matrix) and the relationship among them.

### 3.1. Most Representative Words

First of all, an analysis of word frequency in the focus groups was carried out to examine the most frequently mentioned terms and identify the most relevant among them. The criteria established for calculating word frequency was the selection of the 50 that appeared most often. The list was refined four times, removing empty words and those with no content. 

‘Suicide’ was amongst the most relevant terms that came up in the discourse: being the main topic approached, the professionals used it repeatedly. Most of the times it appears associated with words such as ‘risk’, which, in turn, appeared in its broadest sense with its common meaning of proximity of danger or harm. The term ‘psychiatrist’ was associated by the rest of professionals to the expert of reference when it comes to the management of suicidal behavior, placing special emphasis on the difficulties in accessing them when required for this type of cases. These two, together with the term ‘psychologist’, are the words that were most frequently mentioned by the participants in the study. ‘Primary’ appears associated with ‘care’, since it is another of the professional areas involved in the study, and attention is drawn to the need for communication between primary care physicians, who are the first point of contact for prevention and intervention in cases of suicidal behavior, and psychiatrists. ‘Primary’ also appears in the context of ‘prevention’, the latter being another of the main axes to approach the issue of suicidal behavior. Likewise, in connection with the word ‘program’, they refer to different levels: prevention, primary, secondary, and tertiary. Because it is a clinical context, one of the most frequently used words when talking about people who are at potential risk for suicidal behavior and seek consultation at health centers was ‘patient’. On the other hand, according to the information collected, the term ‘emergency’ appeared in two different contexts: the first was associated with the area of emergency care, and the second it was used to refer to immediate and necessary emergency response actions. As for the tools the different professionals rely on to work with risk behaviors, which include both human and material support, the term ‘resource’ was frequently used. Several of the questions included in the question protocol drawn up for the focus groups were linked to this matter, since one of the purposes was to analyze needs and availability. 

### 3.2. Category Profile

This section describes the relevance of each of the categories that make up the concept at the overall level and for each of the interviewed healthcare groups.

**Figure 3:**
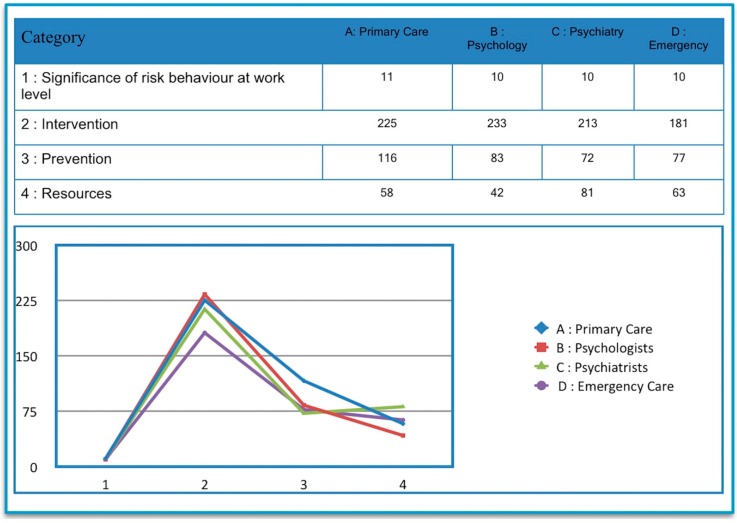
Coded references.

According to the coding analysis, the four groups of professionals taking part in the study agreed that intervention in risky behaviors is first in importance (852 references). Prevention work, with 348 references, was the second main concern of these groups, although it should be noted that psychiatrists attached greater significance to resources and their availability and accessibility than to suicidal behavior prevention, against the results expressed by the other three groups. Nevertheless, is should also be remarked that the difference in psychiatrists’ opinions in terms of prevention and resources was of only nine references. On the subject of current resources, a total of 244 references were gathered. Finally, the lowest number of references was obtained by the “significance of risk behavior at work level” category, with a total of 41 references, although the distribution among the different professional areas is homogeneous (Figure 3).

As regards control of the discursive field during the focus group interviews conducted, commentaries were distributed as follows according to the different professional groups: in the ‘Intervention’ category, the most eloquent professionals were emergency physicians, followed by psychiatrists and psychologists; in the ‘prevention’ category, emergency physicians again made the most comments, followed by psychologists and psychiatrists; in ‘availability of resources’, emergency physicians prevailed once more, closely followed by psychiatrists; and finally, on the subject of ‘significance of risk behavior’, psychiatrists were the professionals who scored the highest in level of participation, followed by emergency physicians. 

#### 3.2.1. Emergency Physicians

For emergency physicians, intervention in suicidal behavior bears the most weight. The ‘Difficulties in intervention’ node is the one with the highest number of codifications and, therefore, the most important for emergency care physicians, with a total of 90 references.

“I don’t think I have the right training in psychiatry to assess many psychiatric patients.” (Reference 4 ‘Difficulties in intervention’—Group 1 Emergency physicians).

“… our work pace in emergency care, which involves an overwhelming demand for care services. I am aware that psychiatric patients require a detailed report and that it is going to take me quite a while if I want to do it properly, as I like to.” (Reference 31 ‘Difficulties in intervention’—Group 2 Emergency physicians).

The next in importance was ‘How intervention in risk behavior is conducted’, with a total of 66 references.

“We are more concerned with the organic condition. If the patient eventually commits another autolytic attempt, or is at risk for suicide or not, is a psychiatric aspect, we always refer them to psychiatrists.” (Reference 1 ‘How intervention in risk behaviors is conducted’—Group 1 Emergency physicians).

“… that is, such case requires organic care and it is given priority more than because of the assessment of risk of autolytic behavior, because the patient’s life and safety come first, and that’s why we don’t proceed otherwise.” (Reference 46 ‘How intervention in risk behaviors is conducted’—Group 2 Emergency physicians).

This category includes contents related to methods of response in cases of risk behavior. The third and fourth place were taken, respectively and according to number of references found in the nodes, by ‘Availability of resources’ (41 references) and ‘Intervention facilitators’ (38 references).

“… there is a specialist on call 24 h that can come.” (Reference 10 ‘Availability of resources’—Group 1 Emergency physicians).

“Nowadays almost every patient requires a multidisciplinary approach. Any patient you might think of, for example a patient with high blood pressure requires the action of several experts.” (Reference 7 ‘Intervention facilitators’—Group 1 Emergency physicians).

Mention should be finally made of the weight given by emergency care physicians to the need to improve response actions, since the ‘How to improve what is being done’ node had 31 references.

“It must be structural improvements. For example, if the problem is more personal, then a better environment is needed.” (Reference 13 ‘How to improve what is being done’—Group 2 Emergency physicians).

#### 3.2.2. Psychiatrists

Just like emergency care physicians, psychiatrists believe intervention in suicidal behavior is of utmost importance, but they also attach significant meaning to prevention of suicidal behavior. It should be noted that the ‘Intervention difficulties’ category includes twice as many references as the second most discussed node, ‘Intervention facilitators’. In this case, as shown in the corresponding figure, 113 references were coded for the first of the most discussed categories and 43 for the second.

“… 90% of what we see are suicidal gestures. The trouble is that there are chances that autolytic behavior as a means to an end might be accomplished. Then, making the right decision in an emergency is very difficult.” (Reference 2 ‘Difficulties in intervention’—Group 1 Psychiatrists).

“… most suicidal people suffer from mental illness, but there is also a part that are people who kill themselves and we didn’t know, or have escaped our attention, or didn’t have any mental illness. So I think that reaching these people is also very difficult.” (Reference 12 ‘Difficulties in intervention’—Group 2 Psychiatrists).

“Psychopharmacological treatment, customizing different treatment plans”. (Reference 9 ‘Intervention facilitators’—Group 1 Psychiatrists).

“Having a nursing service gives one a little reassurance. I feel reassured by knowing that if I’m not seeing the patient that day, or the next, the nurse may see him, or a nurse may pay a home visit and see what has happened, or how he has been feeling, or if he needs something again.” (Reference 24 ‘Intervention facilitators’—Group 1 Psychiatrists).

Other categories on which psychiatrists commented more extensively were ‘Action in prevention’ (38 references), in the field of prevention, and ‘Possibilities in intervention that are not carried out’ (35 references), in the area of intervention.

“I also think that communication between primary and specialized care is fundamental because primary care should act a little as the main filter for problem detection.” (Reference 5 ‘Action in prevention’—Group 1 Psychiatrists).

“We are talking of psychiatrists when psychologists would be the actual point of reference in this matter. Who better than them to assess potential risk for suicide outside the scope of the mentally-ill?” (Reference 1 ‘Possibilities of intervention that are not carried out’—Group 1 Psychiatrists).

#### 3.2.3. Psychologists

The ‘Intervention difficulties’ node yielded the highest number of codes (113), followed by ‘Intervention facilitators’ (79 references).

“There are really quite a lot of impulsive acts that are not based on a perfectly outlined strategy.” (Reference 57 ‘Difficulties in intervention’—Group 1 Psychologists).

“… that scene is very difficult to manage if you don’t have trained and prepared support or reference groups, where you can start working a little.” (Reference 28 ‘Difficulties in intervention’—Group 2 Psychologists).

“It is very important to rely on and be in contact with the patient’s family, and inform the family of the existing risk.” (Reference 1 ‘Intervention facilitators’—Group 1 Psychologists).

However, there are not so many differences between those who work in the area of psychology and the following categories since, although psychologists were much more concerned with prevention (‘Action in prevention’—32 references), the number of references regarding the procedures to be followed to respond to these behaviors (‘How to intervene in risk behaviors’—28 references) and the possibilities to improve intervention (‘Possibilities in intervention that are not being carried out’—26 references) was not much lower, as is the case with ‘Availability of current resources’ (27 references).

“That the patient may come to you at any time regardless of having or not having and appointment, that is, to always leave the door open for them to come, that is the first thing.” (Reference 2 ‘Action in prevention’—Group 2 Psychologists).

“If intervening on the emotional factors involved in the matter is the way of processing feelings. In other words, what we always do.” (Reference 8 ‘How intervention in risk behavior is conducted’—Group 2 Psychologists).

“I think that each case should be looked into individually, which would help to understand and do a little more research to learn some more about how to address this issue. It shouldn’t be dismissed as only attention seeking.” (Reference 8 ‘Possibilities of intervention that are not carried out’—Group 1 Psychologists).

#### 3.2.4. Primary Care Physicians

To complete the analysis of the category profiles, primary care physicians also reported the difficulties they encounter when dealing with these cases (Difficulties in intervention in risk behaviors—115 references), followed, as in most of the mentioned professional categories, by ‘Intervention facilitators’ (63 references).

“I’m not comfortable at all with this condition, I don’t think I’ve got the training to handle it, for many reasons.” (Reference 3 ‘Difficulties in intervention’—Group 2 Primary care physicians).

“I think time is always the main difficulty, because you can’t spend five minutes on this kind of patient, you start to ask and talk…” (Reference 45 ‘Difficulties in intervention’—Group 2 Primary care physicians).

“We already know many of our patients and they come to us frequently…” (Reference 1 ‘Intervention facilitators’—Group 2 Primary care physicians).

“The family, when a patient is at such risk the family knows what must be prevented and watched.” (Reference 7 ‘Intervention facilitators’—Group 2 Primary care physicians).

The third and fourth places were taken by improvement in response (‘How to improve what is currently done’—32 references) and ‘Availability of resources’ (30 references).

“To me, personally, that we be more professional, with less patients. That is, longer consultation time” (Reference 3 ‘How to improve what is being done’—Group 1 Primary care physicians).

“Just as there could be a telephone or situation to detect gender-based violence, I don’t know if there is something similar for this type of behaviors. I’m not aware of it.” (Reference 14 ‘Availability of resources’—Group 1 Primary care physicians).

## 4. Discussion

As it would be expected, the most representative word expressed by the focus groups was ‘suicide’, mainly associated with the word ‘risk’. The next terms that the participants used the most were ‘psychiatrist’ and ‘psychologist’, which reflects the major role played by mental health professionals in the management of suicidal behavior, as well as the frequent link between suicide and mental illness. Conversely, it is interesting to observe how the term ‘primary’ comes up quite often in the course of the discussion in association with different terms such as ‘care’ in the context of primary healthcare as a professional category that is closely linked to suicide, since primary care physicians have the most direct contact with patients and their families and, therefore, would be more qualified for early detection of suicide risk factors. Furthermore, primary care physicians play a major role in primary prevention, ‘prevention’ being the second most frequent term that appears associated with ‘primary’, which reflects the need for intervention in the area of suicide prevention to be delivered at an early stage. Another one of the most recurrent words was ‘resource’, which would point to the need for more human or material tools for suicide prevention. 

An analysis of the findings according to each category profile shows differences among the different professional groups of participants in their perception of the approach and management of suicidal behavior. In general, healthcare professionals consider that attending patients with suicide related behaviors is a huge challenge [27]. The results of this study show that difficulties in intervention in suicidal behavior are the main aspect stressed by the sample of professionals that took part to this investigation. The skills of the different health professionals in the area of suicidal behavior vary widely from one group to another and are closely linked to the individual experience of each of them with this type of intervention [58]. The findings reveal important differences among the groups of professionals. In fact, the main question formulated by general practitioners is knowing clearly how and when to intervene. Thus, training in theoretical models for action and in communication skills would be of the utmost importance [59].

The most remarkable difference concerns the attitude towards risk behaviors of the different professional groups under analysis. This difference is most noticeable between the emergency care group and the rest of the professionals, in particular with mental health experts (psychiatrists and psychologists). 

Specifically, according to professional type, one of the main issues to stress is the broad relationship between primary care physicians and individuals who perform suicidal acts, since their area of expertise entails direct contact with patients in the community. According to a recent study, approximately 80% of the individuals who die by suicide have been in contact with their primary care team during the year before the fatal act [60]. De Leo et al. [31] argue that 90% of the individuals who die by suicide seek help from the healthcare system, especially in the area of primary care, during the three months before their demise. Mention should be made of the fact that primary care physicians are a heterogeneous group of professionals with varying degrees of affinity with mental illness within their clinical practice. This picture reveals the lack of general practitioners in the management of patients with suicidal behavior [61]. One of the noteworthy results of our qualitative study is that most physicians who work in primary care consider that the main obstacles for intervention in the area of suicide are their lack of sufficient skills and knowledge to ensure a successful approach to the issue, a view that is also expressed by emergency medicine physicians. The perception of the existence of failures in approaching and managing patients at risk for suicide expressed by primary care physicians has been previously reported [34,55,62,63,64,65].

Time constraints is another difficulty—according to general practitioners, since it prevents from adequate assessment of patients at risk of suicide. This could be explained by the tight schedule they are expected to follow when seeing patients and could be considered generally inherent to primary care services. Among factors that would make intervention easier for primary care physicians, the most outstanding are their thorough knowledge of their patients, their closeness to them and their possibility of directly accessing patients’ social and family background. These facilitators play a major role in the early detection of risk for suicide and draws awareness to the fact that joint intervention with mental health services should be a key aspect when designing suicide prevention programs. A recent qualitative study stressed the need for primary care physicians to engage the relatives of patients at risk for suicide in the decision-making process [65]. Another study by Bocquier et al. [63] analyzed the abilities of a group of general practitioners in detecting the risk of suicide, yielding a great deal of variation in proper identification, which reveals the need for greater collaboration with mental health experts, as wells as the need for further education and training in how to approach suicidal behavior. Another important aspect in the area of primary care is the availability of and accessibility to the mental health network, in order to count on consultation and referral when needed.

Responses to suicidal behavior in emergency care services are expectedly immediate, paying attention to managing a critical emergency rather than to the identification of the risk for suicide or its prevention. The results of the emergency physicians’ contributions reveal that the involvement of this group of professionals in the management and prevention of suicidal behavior is low, since their priority is to treat the physical injuries resulting from self-harm, considering that the rest of the intervention required in terms of care and prevention falls outside their competence. According to Suokas et al. [66], the skills of emergency care physicians do not vary significantly when there is a psychiatric unit in emergency care, although they generally believe in the need for such a unit and are happy with it. Emergency care physicians’ position of believing that suicide-related behavior is solely the competence of mental health professionals has the obvious consequence of their having less knowledge and skills to manage and prevent it. As a result, the low level of training in the area of suicide of emergency care physicians considerably limits detection of people at risk for suicide when suicidal ideation is not stated as the main reason for seeking medical attention at the emergency department. A recent qualitative research study conducted by Giacchero Vedana et al. [67] using a sample of nursing professionals working in emergency services showed how these professionals express higher levels of negative feelings towards the patient and a sense of lower levels of professional competence in the area of suicidal behavior management which is partly consistent with our results. 

Experts in the area of mental health (psychiatrists and psychologists) believe that the most important aspects with regard to suicide are intervention difficulties. However, against the results yielded by the contributions of emergency and primary care physicians expressing a lack of training and skills in the management of individuals with suicidal behavior, mental health professionals believe that they are sufficiently qualified to address this issue. This is in contrast with a recent study stating that mental health professionals’ main difficulties in addressing suicidal behaviors are related to decision making [25]. Although not associated with training requirements, this is also indirectly revealed by our study, since psychiatrists acknowledge difficulties as regards intervention in and management of suicidal behavior. It should also be emphasized that these difficulties are mostly related to distinguishing between non-suicidal self-injury, not aimed at death, and suicidal behavior, where there is intent to die. In any case, the increasing trend towards the practice of defensive medicine would render decision-making based on patients’ wellbeing as the main target more difficult [68,69]. On the other hand, evidence shows that one out of every three mental health professionals does not regularly ask patients about ideas or thoughts related to suicide [39]. This leads to the conclusion that mental health professionals are perhaps not as aware as they should be of their need for further training and that it could be necessary for them to improve their detection and management skills, regardless of the fact that they might not know it. Either way, we believe that this should not be the main target for improvement in this field. 

The results of this qualitative analysis also reveal the major role played by mental health professionals, especially psychiatrists, in addressing suicidal behavior. In this regard, psychiatrists attach special relevance to the difficulties they have in accessing patients who are outside the mental health network and are at risk for suicide. The high number of people with suicidal behavior who have never been referred to mental health services is quite striking [70,71]. Mental health professionals claim better coordination with primary care as an important factor to detect cases that are not within the mental health network. This result is consistent with a qualitative research study conducted by Roelands et al. [72] involving an analysis of opinions of psychiatrists and emergency physicians, both looking to a greater collaboration between these two professional groups, as well as to a better integration of the mental health network in the area of primary care.

On the other hand, psychiatrists also seem to perceive the need for greater involvement and commitment of psychologists in the area of suicide, strongly believing in the positive effects of psychological therapies to reduce the risk for suicide. A meta-analysis conducted by Calati and Courtet in 2016 [47] confirmed the overall positive effect of psychotherapy interventions in reducing the risk for suicide. Psychiatrists also stress the importance—in everyday clinical practice—of interventions such as pharmacological treatments or community support networks. In fact, community-oriented mental health services register lower suicide rates than traditional mental health services [73].

Professionals in the area of psychology agree with psychiatrists on the difficulties involved in differentiating planned from impulsive acts of self-harm. Psychologists believe that, because of their unpredictable nature, impulsive suicide attempts are more difficult to prevent, thus requiring a more complex intervention on the personality structure of these patients. These professionals believe in the crucial importance of a favorable social and family background towards psychological interventions, with whom to also work independently. Lack of support or referral groups is one of the main problems in the eyes of the psychologists taking part in this study. There is good evidence of the effectiveness of psychosocial interventions in suicide prevention, and in recent years we have witnessed the development of new therapies focused on the family and the environment of the individual at risk for suicide [74,75,76,77,78,79]. In agreement with psychiatrists, psychologists believe that community support networks would facilitate suicide prevention and contribute towards patient adherence to psychotherapeutic interventions, while also enhancing the chances of intervening during crises and being able to identify changes in behavior that may hint at a potential risk for suicide. The results of a study by Gilat et al. [80] using online support groups suggest that these groups allow individuals who have engaged in suicidal behavior to create an atmosphere where they can find emotional support and alternatives to suicide to address their problems.

This study has the strength of including participants with wide experience identifying and managing suicidal behaviors because this is part of their daily clinical practice. In addition, the whole methodology and analysis of results has been carried out by researchers related to the University of Salamanca with vast experience in qualitative analysis. On the other hand, we do not know other studies where the perceptions of the different groups of health professionals who have the most contact with the problem of suicide have been analyzed and compared. This provides a great value to the results of our research, which will contribute to the development of a suicide prevention plan specific to our region. Other potential benefits could be derived from this investigation. However, there are a number of limitations to this study: (a) This is a small sample size of healthcare professionals. Therefore, these findings must be interpreted with caution, as they may not be generalizable to a larger population. It must be borne in mind that, as in all qualitative research, the results are subject to the context of the study. For its generalization, a scale should be built based on the results obtained, thus going towards methodological complementarity, mixed models, according to the current projection of research in the health sciences. (b) All of participants are involved in the same geographical area (health network of Zamora) so, many of the results and points of view of the participants can be influenced by the specific features of the mental health network of Zamora. However, there are few differences between the statistics of suicide in Zamora vs. the others health areas of Spain, and there are not specific features of the mental health network. (c) The participants involved in the study have not chosen or recruited in a randomized way, but taking into account the type of analysis (qualitative) was more important to choose people representative of all points of view of the health system and obtain proposals from them, than do the study with a representative sample. The interest was to know the different points of view of health professionals about the unmet needs and proposals for improving and so, the recruitment was addressed for achieving this goal.

## 5. Conclusions

The conclusion that can be drawn from these findings is that there are needs to be met and policies to be developed to improve the care of people at risk for suicide. The following points summarize desirable improvements in each area of the healthcare network involved in the management and treatment of suicidal behavior.

### 5.1. Primary Care Physicians

- Need for more time to address patients at risk for suicide.
- Easier access to and communication with the mental health network.
- Availability of immediate or within 24 h referral.
- Lack of training in the management of suicidal behavior.

### 5.2. Emergency Care Physicians

- Lack of awareness of their role in the detection of risk for suicide in patients who seek attention at emergency care facilities for reasons of general somatic issues.
- They focus their response on handling the risk for death to later refer the patient to psychiatric services.

### 5.3. Mental Health Care Physicians

- High demand, especially in self-harming behaviors that require a specific approach.
- Give more priority to psychotherapeutic interventions and improve the availability and role of clinical psychologists in the management of suicidal behavior.
- Need for the implementation of specific programs to address suicidal behavior: group therapy, etc.
- Accessibility should be an important part of intervention.
- Importance of the role of a community support network, especially involving home care by nursing professionals.

Improvement in coordination with primary care for the detection of cases that are not within the mental health network.
